# Machine Learning Analysis of RNA-seq Data for Diagnostic and Prognostic Prediction of Colon Cancer

**DOI:** 10.3390/s23063080

**Published:** 2023-03-13

**Authors:** Erkan Bostanci, Engin Kocak, Metehan Unal, Mehmet Serdar Guzel, Koray Acici, Tunc Asuroglu

**Affiliations:** 1 Department of Computer Engineering, Faculty of Engineering, Ankara University, 06830 Ankara, Turkey; 2 Department of Analytical Chemistry, Faculty of Gülhane Pharmacy, University of Health Sciences, 06018 Ankara, Turkey; 3 Department of Artificial Intelligence and Data Engineering, Faculty of Engineering, Ankara University, 06830 Ankara, Turkey; 4 Faculty of Medicine and Health Technology, Tampere University, 33720 Tampere, Finland

**Keywords:** transcriptomics, RNA-seq, machine learning, deep learning, classification, cancer prediction, exRNA

## Abstract

Data from omics studies have been used for prediction and classification of various diseases in biomedical and bioinformatics research. In recent years, Machine Learning (ML) algorithms have been used in many different fields related to healthcare systems, especially for disease prediction and classification tasks. Integration of molecular omics data with ML algorithms has offered a great opportunity to evaluate clinical data. RNA sequence (RNA-seq) analysis has been emerged as the gold standard for transcriptomics analysis. Currently, it is being used widely in clinical research. In our present work, RNA-seq data of extracellular vesicles (EV) from healthy and colon cancer patients are analyzed. Our aim is to develop models for prediction and classification of colon cancer stages. Five different canonical ML and Deep Learning (DL) classifiers are used to predict colon cancer of an individual with processed RNA-seq data. The classes of data are formed on the basis of both colon cancer stages and cancer presence (healthy or cancer). The canonical ML classifiers, which are k-Nearest Neighbor (kNN), Logistic Model Tree (LMT), Random Tree (RT), Random Committee (RC), and Random Forest (RF), are tested with both forms of the data. In addition, to compare the performance with canonical ML models, One-Dimensional Convolutional Neural Network (1-D CNN), Long Short-Term Memory (LSTM), and Bidirectional LSTM (BiLSTM) DL models are utilized. Hyper-parameter optimizations of DL models are constructed by using genetic meta-heuristic optimization algorithm (GA). The best accuracy in cancer prediction is obtained with RC, LMT, and RF canonical ML algorithms as 97.33%. However, RT and kNN show 95.33% performance. The best accuracy in cancer stage classification is achieved with RF as 97.33%. This result is followed by LMT, RC, kNN, and RT with 96.33%, 96%, 94.66%, and 94%, respectively. According to the results of the experiments with DL algorithms, the best accuracy in cancer prediction is obtained with 1-D CNN as 97.67%. BiLSTM and LSTM show 94.33% and 93.67% performance, respectively. In classification of the cancer stages, the best accuracy is achieved with BiLSTM as 98%. 1-D CNN and LSTM show 97% and 94.33% performance, respectively. The results reveal that both canonical ML and DL models may outperform each other for different numbers of features.

## 1. Introduction

Colorectal cancer is among the most common cancers around the world. It has high incidence and mortality rate with increasing trend. Many factors, such as smoking and alcohol consumption, could contribute to incidence of colorectal cancer. Currently detection methods for colorectal cancer, such as colonoscopy and fecal occult blood test, have various disadvantages. These disadvantages are lower sensitivity and specificity and also bleeding problems. Moreover, many patients can be diagnosed at late stage of colorectal cancer. Therefore, there is a great demand for rapid and reliable detection methods for diagnosis and prognosis of colorectal cancer.

In recent years, attention has been drawn to omics technologies in life sciences and clinical analysis. These techniques provide essential information about the pathogenesis of diseases at metabolite, protein, and transcriptome level. Transcriptomics is the general analysis of organism’s transcriptome, in other words, the sum of all RNA transcripts. Transcriptomics have been used to understand nature of diseases and to find diagnostic and prognostic biomarkers. Moreover, high throughput RNA-seq data could provide an opportunity to analyze hundreds of transcripts for a complete view of the expression dynamics of diseases.

ML, which is a branch of artificial intelligence, provides computers the ability to create models from data. It has been used in many fields of healthcare [1,2,3]. In particular, using health records in ML systems provides vast opportunities to answer clinical problems [4,5,6].

Another promising area in healthcare is omics technology [7]. Recent developments in genomics, transcriptomics, proteomics, and metabolomics have opened new opportunities for personalized and precision medicine. Omics have been used to understand disease mechanism, treatment efficacy, and lifestyle interventions for diseases [8]. In the last decade, the amount of data produced in omics technologies has increased exponentially. The idea of integrating omics data with ML methods is to provide more comprehensive understanding of biological systems. In particular, evaluation of clinical omics studies has opened a new aspect in diagnosis and prognosis of diseases [9,10,11].

Transcriptomics is the analysis of global transcriptome, which is the complete set of RNA transcripts [12]. It provides an opportunity to analyze the expression level of transcripts for understanding physiological or pathological conditions. Transcriptomics have become one of the most utilized approaches that analyze human diseases at molecular level by using high-throughput methods (RNA array or RNA-seq) [13]. The expression analysis of transcripts is used to find biomarkers and therapeutic targets for many diseases [14,15]. In recent years, ML methods have been applied to transcriptomics data in various clinical studies, and results have provided essential information for future clinical approaches. However, this integration is not easy because transcriptomics analysis is expensive and ML systems require large sample sizes to thrive in prediction tasks. Therefore, previously published studies evaluated ML systems to obtain more information from biological datasets.

**Figure 1:**
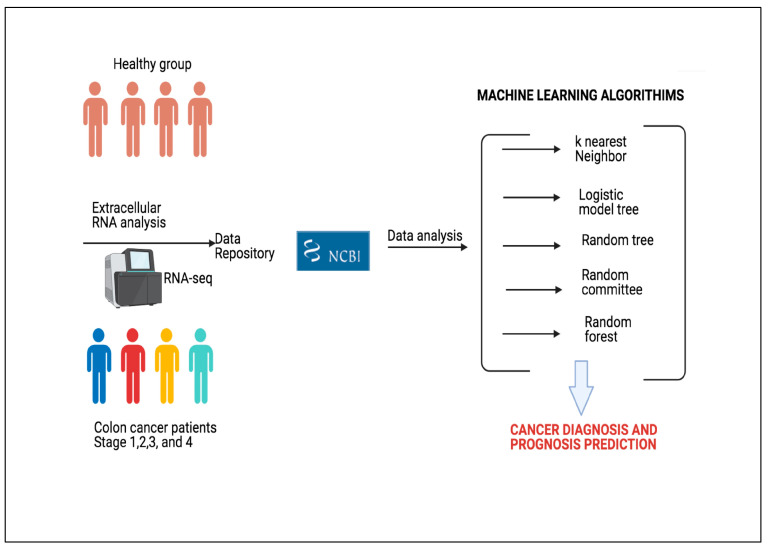
The general framework of proposed approach. (Illustrations by the authors).

In the present study, we focused on evaluation of transcriptomics analysis of circulating EVs in ML systems to predict colorectal cancer and to classify cancer stage. EVs, such as exosomes, play an important role in intercellular communications. They carry various types of bioactive molecules, including membrane proteins, lipids, RNAs, and DNA [16]. Their components are highly variable depending on the cells of origin. In cancer research, attention has been drawn to EVs because tumor-derived EVs contain unique materials (such as RNA and protein) for diagnosis and prognosis of cancer [17]. Yuan et al. analyzed RNA profile of plasma EVs in healthy and cancer patients [18]. Their aim was to find novel RNA based biomarkers for diagnosis and prognosis of various cancer types. Their study was one of the largest scale studies on various cancer types. Herein, we analyzed their dataset using different ML and DL approaches and tested the capability of our proposed approach to be used as a diagnostic and prognostic tool. We believe that our approach could contribute to further studies regarding integration of omics data with ML methods. The general framework of the study is given in Figure 1.

Our first hypothesis is that DL algorithms will yield higher results in terms of accuracy than canonical ML algorithms since DL algorithms have more parameters and higher learning capacity than ML algorithms.

The second hypothesis is in regard to exRNA transcripts that are used to feed both DL and canonical ML algorithms. Since miRNAs are the most abundant exRNA transcripts in homo sapiens and it is known that they are relevant to various cancer types, it is expected that miRNAs will be selected as more informative than other exRNA transcripts by a feature selection algorithm. 

The third hypothesis is that utilizing more informative exRNA transcripts selected by a feature selection algorithm rather than utilizing all attributes (exRNA transcripts) as inputs to feed the algorithms can improve the performance of the models.

To summarize, the aim of the study is to develop canonical ML and DL models for predicting colon cancer and classifying the cancer stage. The experimental results reveal that both ML and DL models show promising performance. In addition, the results of McNemar’s test indicate that a statistically significant difference exists among models. The contribution of this study is fourfold. The first one is the comparison of canonical ML and DL algorithms. According to the experimental results, DL models have higher accuracy than canonical ML models for both cancer prediction and cancer classification. The second one is the development of DL architectures. In the study, instead of using a pre-trained model, all DL models are constructed from scratch and hyper-parameters are optimized by utilizing the GA, which is a meta-heuristic optimization technique. The third one is the feature selection. According to the results, instead of using all attributes, selecting some attributes that are more informative than others for the training phase can increase the accuracy of a model. By reducing the dimension of the feature space, the training time of a model is also shortened. The fourth contribution of the study is the uncovering of exRNA transcripts that may be determinative in colon cancer. Among 493 exRNA transcripts/attributes, 49 of the most informative exRNA transcripts belong to the mature miRNA category. The experimental results reveal that the RF algorithm fed by the most informative 30 and 40 exRNA transcripts outperformed other canonical ML algorithms in terms of accuracy for classifying colon cancer stage. 

The remainder of the article is organized as follows. Section 2 includes the literature review. In Section 3, information about the data, the data augmentation method, min–max normalization, the attribute selection method, the cross-validation technique, canonical ML and DL algorithms, GA that is used for optimization, evaluation metrics, and statistical tests are presented. Section 4 presents the experimental results, discussion, and practical applicability. In Section 5, the article is concluded.

## 2. Related Works

The literature presents different usage of ML algorithms on transcriptomic data. Pantaleo et al. used blood transcriptomics data to train ML algorithms for early detection of Parkinson’s disease (PD) [19]. In this study, a dataset of 550 samples is used to train and test ML models. A feature selection mechanism, which includes RF, is used to reduce the dimensionality. The selected features were used to train the eXtreme Gradient Boosting (XGBoost) model with 10-fold cross validation method. This cross-validation phase is repeated 20 times with different seeds to obtain the best tuning parameters for RF algorithm. The average accuracy of the XGBoost model was 69.3%.

Nalls et al. designed a model for early diagnosis of PD using Linear Regression [20]. The training dataset contained information of 532 individuals, 367 of which had PD and 165 of which were healthy. In that study, the test set included 1086 samples, 825 of which had PD. They used area under the curve (AUC) and sensitivity as the evaluation metrics, which were 0.923 and 0.834, respectively. 

Hamey and Göttgens used different ML algorithms to evaluate the similarity of single-cell transcriptomes to the hematopoietic stem cells [21]. In that study, kNN, Linear Regression, Multilayer Perceptron (MLP), RF, and Support Vector Machines (SVM) models were trained with fivefold cross validation. Among these models, MLP and SVM generated the best results regarding hscScore, which defines similarity to gene expression profiles of validated hematopoietic stem cells.

Akter et al. used ML models to diagnose endometriosis using RNA-seq and DNA methylation [22]. In that study, the candidate biomarker genes were determined using various techniques, and then four different supervised ML methods, namely Decision Tree (DT), Partial Least Squares Discriminant Analysis (PLSDA), SVM, and RF, were trained. The results were evaluated using different metrics, including accuracy, sensitivity, precision, etc. DT was the overperforming technique among the four ML methods, with 89% accuracy.

Sharifi et al. employed tree-based ML methods with meta-analysis to identify transcriptomic biosignature of mastitis disease [23]. These tree-based models, that included RF, successfully detected the best combination of genes as biosignature which helped to diagnose the disease early.

DL models have also been used in cancer diagnosis studies. Balaha et al. designed a model for early diagnosis of breast cancer using ultrasound data [24]. The study presented a hybrid model using both CNN and GA. The Transfer Learning method, which included tuning popular pretrained CNN models, were employed. GA was used for parameter optimization and learning. The dataset contained images of breast ultrasound and augmented during the training process. For evaluation metrics, they used loss, accuracy, F1-score, precision, recall, specificity, and AUC. Among the pretrained models, Xception showed the best performance, achieving over 90% accuracy and F1-score.

Anaraki et al. proposed a method which used CNNs and GA to classify different stages of brain tumor [25]. The dataset contained brain MR images of individuals who were healthy or suffered from different level of cancer. In that study, they designed an evolving CNN structure rather than existing pretrained models. In the phase of data augmentation, a straightforward method, which included rotation, translation, and scaling, was used. After this step, a total of 16,000 MR images, which included 8000 healthy and 8000 with tumor, were obtained. In the training step, different parameters were used to evolve the CNN with GA, including, but not limited to, number of convolutional, max pooling, fully connected, and dropout layers. In addition, Bootstrap Aggregating was employed to decrease the generalization error. This study showed average accuracy of 90% after seven generations of GA. 

Dweekat and Lam presented a hybrid system with GA, MLP, and Principal Component Analysis (PCA) to predict cervical cancer [26]. In that study, PCA was used for feature transformation, MLP used as classification model, and GA used to optimize the hyperparameters of MLP. The proposed method outperformed existing techniques with fivefold cross validation, with 96% accuracy. 

Resmini et al. purposed an ensemble method with GA and SVM to diagnose breast cancer using thermographic data [27]. The reason for using thermographic data in this study was the low measurement cost. The classification system included three stages. In the first stage, best model was selected using GA. In the next stage, GA was also employed to select features. The classification was performed at the last stage. They achieved promising experimental results, with 97% accuracy and 94% AUC. 

Consiglio et al. used Fuzzy Rules with GA to separate ovarian cancer and other ovarian diseases [28]. Here, GA was employed for the feature selection phase with if–then rules. The purposed method can help to discover changes in the selected genes over the distinguished classes. The dataset in that work contained 21 samples with 45,000 genes that corresponded to the features. After the feature selection phase, a 9000-feature dataset was obtained. The classification task was performed using a Fuzzy-Rule-Based System which included a form of if–then rules. A different set of parameters was prepared for GA, which included 100 to 400 individuals. They reported 100% accuracy on the dataset. 

Ali and Saeed proposed a system that included hybrid filter and GA to reduce the feature space of microarray data, which generally has high dimensions and causes slow performance on ML algorithms [29]. In the initial step of the study, information gain, information gain ratio, and Chi-square were used for feature selection of cancerous microarray datasets. The next step included employing GA to optimize the feature selection process. The dataset with selected features was used to train different ML algorithms, including DT, kNN, RF, and SVM. Accuracy, recall, precision, and f-measure were used as evaluation metrics. Experimental results indicated that the proposed approach increased the performance of all models regarding all evaluation metrics.

The literature presents many different approaches for diagnosis of colon cancer using ML/DL methods. Jiang et al. designed CNN- and ML-based prediction systems for colon cancer [30]. In that study, the system was designed only for stage III of colon cancer and used hematoxylin-and-eosin-stained tissue slides. 

Gupta et al. [31] demonstrated the prediction capabilities of different ML algorithms using information that contained histopathology reports, intra-operative findings, history taking, and chart records. The dataset was not augmented in the training stage and was used as it was. The study focused mainly on stage prediction of the colon cancer and used RF, AdaBoost, SVM, MLP, and kNN as classifiers. The Recursive Feature Elimination method was used as the feature selection algorithm. The accuracy results for the RF, which was the overperforming algorithm, were 74% and 90% when taking only the tumor size as a prognostic factor and taking Tumor Aggression Score as a prognostic factor, respectively.

Masud et al. presented a framework to diagnose lung and colon cancer tissues using DL [32]. In that study, a lung and colon cancer histopathological image dataset, which contained 25,000 color images with 5 different classes was used. The classification was performed using a CNN. The framework demonstrated a maximum accuracy of 96%.

As can be seen from previous studies, ML and DL algorithms were fed by thermographic, MRI, or CT images. We attempted to fill the gap in the literature by utilizing transcriptomic data of individuals. Another gap in the literature is that the hyper-parameters of DL architectures are not optimized. We attempted to contribute to the literature by building DL architectures from scratch and optimizing DL hyper-parameters with the meta-heuristic GA to be utilized in colon cancer prediction and classification. Furthermore, the collection of data is more straightforward and preserves the life quality of the patients compared with classical methods. Our proposed approach offers the benefit of determining disease progression simply by re-obtaining a patient’s exRNA transcript values, without subjecting the patient to procedures that could impact them physically or mentally.

## 3. Materials and Methods

### 3.1. Study Subjects and RNA-seq Analysis

In this work, the dataset from Yuan et al.’s study is used, which has GEO database accession number of GSE71008 [18]. This study contains 50 healthy subjects and 100 patients with colorectal cancer (n = 25 for each of stages I-IV). The RNA-seq analytical pipeline eRNA (v1.2) was used for the data analysis, including raw data extraction, trimming, sequence alignment, and read count scaling. In Yuan et al.’s work [18], they used various databases, including miRNA, piwiRNA, siRNA, and FLJ human cDNA. In addition, miRNA isoform analysis and exRNA stability analysis tests were carried out. They used normalized RPM values for comparison between healthy and cancer patients. In this work, similar workflow was employed to analyze RNA-seq data. Normalized RPM level of RNA transcript was utilized for ML systems. In addition, log2-transformed RPM cut off value was determined as 5 for reliable analysis.

### 3.2. Data

**Figure 2:**
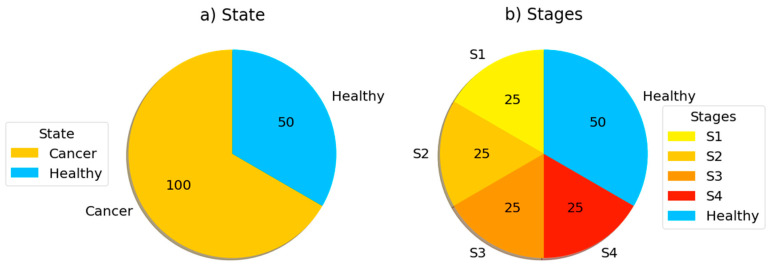
The distribution of data (**a**) for prediction and (**b**) for classification. (Illustrations by the authors).

A total of 150 subjects were separated as shown in Figure 2. As can be seen in Figure 2a, 100 of 150 subjects were cancer patients at a certain level. As seen in Figure 2b, 100 patient individuals were equally divided in 4 stages of the disease. 

The ML methods generally require large datasets. The data we used in this study were from 150 subjects and may be insufficient for this type of work, and an augmentation of the data was required to achieve a satisfactory performance of ML classifiers [33]. For this purpose, the data were augmented to include 300 samples. Later, considering the large number of features compared with the sample number of the data, a certain number of features were selected, considering that some features may be more important than others. In addition, it is important to note that selecting features reduces the complexity of the data and shortens the training time [34]. 

For data augmentation, the Synthetic Minority Over-sampling Technique (SMOTE) algorithm was utilized in the study [35,36]. SMOTE was originally developed for imbalanced datasets to oversample the minority class. However, it can also be used to oversample the whole dataset. SMOTE oversamples the minority class by generating synthetic data by working on feature space. This method oversamples by taking every minority class example into account and presenting synthetic examples and joining nearest neighbors to that class. The nearest neighbor count depends on the size of the oversampling process. The first step of generating synthetic examples is calculating the difference between the feature vector of current example and its nearest neighbor. The second step includes multiplying the calculated difference by a randomly generated number between 0 and 1. In the third step, the calculated vector is added to the feature vector of the current example. 

In our study, first, for a randomly selected healthy sample, 50 augmented healthy samples were generated, while 50 randomly selected cancerous samples were used. Therefore, a total number of 100 healthy samples were obtained. Secondly, for a randomly selected cancerous sample, 100 augmented cancerous samples were generated, while all healthy samples were used. As a result, a total number of 200 cancerous samples were achieved. In total, the size of the dataset was increased to 300 samples while keeping the imbalanced ratio. 

On the data, normalization was applied to reduce the effect of outliers and guarantee that all attributes have the same scale for both canonical ML and DL algorithms. In our study, min–max normalization was used for data normalization process. Min–max normalization can be seen in the following Equation (1):

(1) $$x^{\prime }=\frac{x-min\left(X\right)}{\max\left(X\right)-min\left(X\right)}\left(new\_max\left(X\right)-new\_min\left(X\right)\right)+new\_min\left(X\right)$$

In the equation above, $x^{\prime }$ and x represent the new normalized and the current values of the attribute, respectively, whereas min(X) and max(X) represent the current minimum and the current maximum values, respectively, in the related attribute column of all samples; new_min(X) and new_max(X) represent the new minimum and the new maximum values, respectively, in the new normalized range.

In this study, standard [0–1] min–max normalization is applied for canonical ML algorithms, while [0–255] min–max normalization is preferred for DL algorithms. The reason for this choice is that CNN architecture accepts images as input (in the experiments 1-D CNN is fed by gray scale images). In addition, LSTM and BiLSTM algorithms were fed by input values having a range between 0 and 255.

In the dataset that is used in the study, there existed 493 attributes for each sample. To observe the effect of the number of attributes that will be given as inputs to ML and DL algorithms on the performance, a feature selection algorithm was applied. Feature selection can help to reduce dimensionality and, therefore, reduce computational load of ML frameworks. In addition, by selecting relevant features, accuracy of predictions can be increased [37]. Simply, the algorithm calculated the information gain (*IG*) for each attribute. *IG* can be defined as expectation of entropy reduction while splitting the samples according to an attribute. In other words, IG determines how much information an attribute supplies about a class. Therefore, the higher value of *IG* of an attribute, the more informative it is. *IG* can be calculated as in the following Equations (2) and (3):

(2) $$IG\left(C,X\right)=Entropy\left(C\right)-\underset{x\in X}{\sum }\frac{X_{x}}{X}*Entropy\left(X_{x}\right)\;$$

(3) $$Entropy=-\sum_{i=1}^{c}P\left(x_{i}\right)log_{2}P\left(x_{i}\right)$$

In the equations above, C represents the target or class, X represents the attribute vector, and x represents each value of the attribute vector X. While calculating entropy, c represents the number of the cases of the target or briefly the number of classes. Finally, $P\left(x_{i}\right)$ represents the probability of a value occurring in the target data. 

For the experiments, *n* attributes with the highest IG values were selected to feed the algorithm for training process. In our study 10, 20, 30, 40, and 50 attributes having the highest IG scores were selected, and all experiments were conducted by using these attributes. In addition, the experiments were repeated and compared on the basis of performance by including all attributes. 

**Figure 3:**
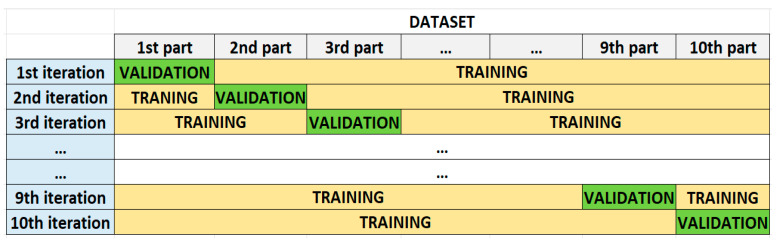
Stages of 10-fold cross validation. (Illustrations by the authors).

In all experiments, to calculate the performance of the models, based on the evaluation metrics, the 10-fold cross validation technique was employed. According to this technique, the dataset was split into 10 equal parts while maintaining the class ratio. In the next step, the first part was excluded, while the remaining part was used to train the ML or DL algorithm. After the training phase, the obtained model was validated with the excluded part. These processes were repeated until all parts were used to validate the models (Figure 3). To evaluate the final accuracy of a model after 10-fold cross validation, the accuracy results of all folds were taken into consideration. The final accuracy was calculated by averaging the accuracy results of the 10 folds.

### 3.3. Machine Learning Analysis

The popularity of ML has tremendously increased over the last decade. This increase has enabled ML to be applied to increasingly more areas. One of the most important applications of ML is the prediction of diseases. By analyzing the data obtained from individuals, the probability of disease can be predicted with high accuracy. 

The literature presents different approaches of ML in medical domain and disease prediction. In this study, we used 5 canonical ML approaches to predict whether an individual has colon cancer. The selected methods are kNN, LMT, RT, RC, and RF. All canonical ML algorithms were employed with default parameters (Table 1). In addition, DL algorithms were utilized to predict the stage of the cancer and whether an individual has colon cancer. 1-D CNN, LSTM, and BiLSTM DL algorithms were applied in the study and optimized.

kNN is the one of the most used approaches of ML. This supervised learning method was presented in 1967 by Cover and Hart [38]. This approach classifies a sample by looking at its previously classified neighbor samples and is independent of the hidden joint distribution on other samples and their classification. The literature has different applications of kNN on cancer diagnosis, particularly in breast cancer [39,40,41,42].

LMT is a supervised classification algorithm, which is the combination of two learning approaches with complementary superiority and weakness: DT and Logistic Regression [43]. The LogitBoost algorithm is used to generate a logistic regression model at each node of these classification trees with logistic reduction functions on their leaves. In this way, it is ensured that the child nodes contain information about the main nodes and that probability estimates are formed for each class. The resulting model is simplified by dividing it according to C4.5 criteria. LMT algorithm is used for disease classification [44,45] and predicting cancer and cancer proteins [46,47]. 

RT is a DT-based supervised classifier that randomly selects the k number of attributes at each node [48]. The algorithm has no pruning to decrease the error and is very effective on classification and regression tasks. The classifier depends mainly on the single model tree and Random Forest [49]. Previous studies demonstrate that RT classifier is easy to implement, effective, and does not overfit [50,51].

RC is an ensemble classifier which uses base classifiers with the same data but a different number of seed values to make a predictions separately [52]. The algorithm forges final prediction by averaging the results of these individual base classifiers [53]. In the literature, RC is used in disease prediction tasks [54].

RF is another widely used classifier that utilizes a group of unpruned DTs and is accurate on large volumes of data in classification and regression tasks [55]. This group of DTs is built from a training data set and determines the output. Each DT in this group is a separate classifier and has its own predictions from a sample. This algorithm combines all the results from DTs to decide the final prediction [56]. The RF classifier is used to predict different cancer types, such as esophageal [57], breast [58,59], prostate [60], colorectal [56], lung [61], and cervical [62].

LSTM networks are an upgraded version of recurrent neural networks (RNN) [63]. In recent years, they output better classification results when compared with other DL networks on various research areas, such as time series and genome data [64,65]. In order to comprehend LSTM structure, RNN structure needs to be defined. RNNs are neural networks that also have memory and are able to recall all the information that is sequentially captured in the previous element. In other words, RNNs are an efficient way to use data from relatively long series since they perform similar tasks for each element in the series, with output dependent on all previous computations. A network with a feed-forward architecture and an extra cyclic loop is considered as RNN. By using this cyclic loop, RNN carries information throughout the network one time step to the next one. A form of short-term memory, cyclic loops are used to store and retrieve historical data throughout time steps. 

An RNN that learns temporal patterns estimates the current time-step by using the prior state and the present state. However, RNN architectures come with a disadvantage—vanishing gradients. The issue of vanishing gradients arises when recurrent neural networks are required to learn long-term relationships in time steps. For this requirement, the gradient vector increases or decreases exponentially as it propagates through multiple layers of the RNN to learn long-term dependencies over time steps. LSTM aims to solve this issue. In order to tackle vanishing gradient problem, LSTM uses memory blocks instead of traditional RNN units [65]. Its main advantage over RNNs is that it incorporates a cell state to store long-term states. An LSTM network can remember and connect information from the past to current information [64]. An updated version of LSTM called BiLSTM has emerged in recent years [66]. This architecture enables LSTM to analyze input data both forward and backwards. It actually adds two layers of memory cells to analyze data on both ways. The binding process of hidden states of backward and forward layers creates the representation of input data [67].

1-D CNN is a modified version of CNN DL model [68]. In this version, one dimensional convolutional layers and sub-samplings are used to build feature space [69]. The one-dimensional convolution patch is handled by a number of convolution and pooling layers in the model, which extract features from one-dimensional input using a local receptive field and shared weights. These shared weights adjust the number of training parameters to be less than traditional CNN architectures. Through the use of several convolution filters, feature maps in the convolution and sub-sampling layers derive discriminant feature representations from many input vector segments. The 1-D CNN classifier is constructed with sample class information in the training process, and the gradient descent algorithm is utilized for adjusting network parameters [70]. 

The general structure of CNN consists of convolution, pooling, and a fully connected layer [69]. In the convolution layer, several convolution filters are employed to extract representative information from the raw data. Neurons are connected locally, thus reducing calculation load. In pooling layer, a process called sub-sampling is used to obtain more detailed feature maps at a lower resolution. The fully connected layer generally comes before the output layer to forward features to final classification phase [71].

The experimental setup for canonical ML algorithms can be seen in Table 1, Table 2, Table 3 and Table 4, which indicate the hyper-parameters of the proposed 1-D CNN architecture. Table 5 and Table 6 show hyper-parameters to build LSTM and BiLSTM architectures from scratch. Therefore, the results can be reobtained for each model by utilizing the optimized hyper-parameters.

All canonical ML and DL algorithms have some advantages and disadvantages. Their performances are closely related to the utilized dataset. The advantages and disadvantages of the algorithms utilized in this study are explained briefly. In our study, kNN is chosen since it is easy to implement and it makes no assumptions about the data. However, it has a disadvantage in dealing with imbalanced data. LMT algorithm is expected to provide accurate results since it combines decision tree and logistic regression algorithms. In contrast, due to its high computational cost, it is not a preferred algorithm. The advantage of RC is that it takes into account the results of different classifiers. Likewise, this situation can lead to a disadvantage. If the majority of the classifiers make an incorrect prediction, the algorithm’s prediction will also be incorrect. RF’s advantage is that it is composed of uncorrelated decision trees. In other words, the trees that form the forest are not similar. Therefore, the algorithm has a high generalization capacity and handles imbalanced data. Nevertheless, if a dataset does not have some informative attributes, prediction performance of RF will suffer. As with RF, the performance of the RT algorithm directly depends on whether there are some informative attributes in the dataset. Consequently, if a dataset is an imbalanced one and some of the attributes have importance, it will be more likely expected that RF yields better accuracy than other ML algorithms. 

CNN is chosen because it exhibits high performance when classifying images. Since an image is a matrix, we can build a model using CNN architecture if we express each sample as a 1-D matrix. LSTM and BiLSTM are efficient in processing sequential data. In addition, if we have 1-D matrices as inputs, we can feed these algorithms. All DL algorithms utilized in the study suffer from the training time to build a model.

To compare the algorithms, some evaluation metrics are needed. One metric is not sufficient to reveal the superiority of an algorithm. To support the accuracy of the algorithms, statistical tests are applied on the results. In this study, the Kappa statistic and McNemar’s test were utilized to validate the results. 

While experimenting with DL algorithms, values of some hyper-parameters needed to be optimized. Therefore, for each DL algorithm, GA, a meta-heuristic approach, was utilized for optimization. 

**Figure 4:**
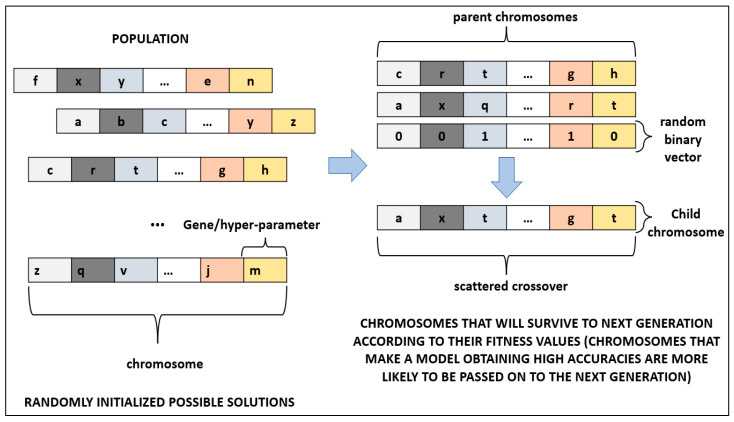
Process of scattered crossover in GA. (Illustrations by the authors).

GA is a meta-heuristic search algorithm that mimics the evolutionary process, having the principle of the survival of the fittest. Especially in cancer diagnosis, GA has a wide range of use [27,72]. In this study, GA was utilized to optimize hyper-parameters of DL algorithms. Each possible solution was represented by a chromosome in GA. A chromosome is composed of genes that represent the hyper-parameters to be optimized of a DL architecture. All chromosomes form a population where the optimal chromosome, which satisfies the fitness function, is attempted to be found. Firstly, a population is initialized randomly. Secondly, fitness value of all chromosomes is evaluated in the population. Thirdly, the parent chromosomes that will form the next generation are chosen. Crossover and mutation operations are applied on chosen chromosomes. The third step is repeated until a stopping criterion is met. Some of the chromosomes pass on the next generation directly; these chromosomes are called elites. In our study, the number of generations was selected as 100, and it was used as the stopping criterion. The percentage of elites was selected as 5% of the population. The crossover operation that determines the fraction of the next generation was applied on 80% of the population. The rest of the population was mutated while surviving to the next generation. Since GA does not guarantee the global minimum, a large population size of 200 was selected to reduce the probability of obtaining a local minimum while increasing the run time of the algorithm. To produce children chromosomes, scattered crossover was utilized for crossover operation (Figure 4). In scattered crossover, after selecting the parent chromosomes, a randomly created binary vector determines the genes of the child chromosome (Equation (4)).

(4) $$g_{i}\left(C_{c}\right)=\{\begin{array}{c} g_{i}\left(C_{p2}\right),\;b_{i}=0\\ g_{i}\left(C_{p1}\right),\;b_{i}=1 \end{array}$$

In Equation (4), $g_{i}$ represents the *i*th gene in the child chromosome ($C_{c}$) and parent chromosomes ($C_{p1}$ and $C_{p2}$), while $b_{i}$ represents the *i*th value in the random binary vector. 

**Figure 5:**
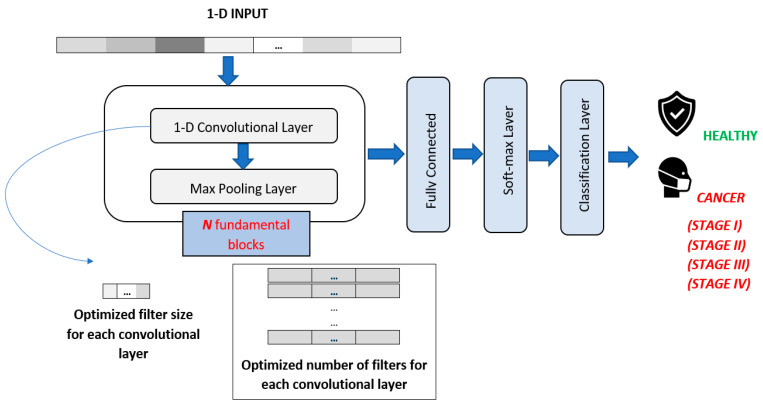
Proposed 1-D CNN architecture. (Illustrations by the authors).

For 1-D CNN DL, hyper-parameters, such as filter size and number of filters, were optimized for each convolutional layer by applying GA. General structure of the 1-D CNN architecture is shown in Figure 5. 

The number of convolutional layers to be added in the 1-D CNN architecture was determined by the size of the input (attributes). Therefore, for each number of attributes (10, 20, 30, 40, 50, and all attributes that form the feature vector) different numbers of convolutional and max pooling layers existed in the related 1-D CNN architecture (Table 2). For each convolutional layer, the stride parameter was selected as 1 and zero padding was applied, when necessary, to make the output as the same size as the input. After each convolutional layer, there existed a max pooling layer in the architecture. Max pooling layers halve the size of the input to perform down sampling. To ensure that, the stride parameter and pool size parameter were selected as 2 and 3, respectively, and zero padding was applied, when necessary. Consequently, different numbers of convolutional and max pooling layers were added according to the size of the input in the architecture until the output size was 1. 

Optimized values by applying GA for 1-D CNN architecture can be seen in Table 3 and Table 4 for both cancer prediction and cancer stage classification. 

**Figure 6:**
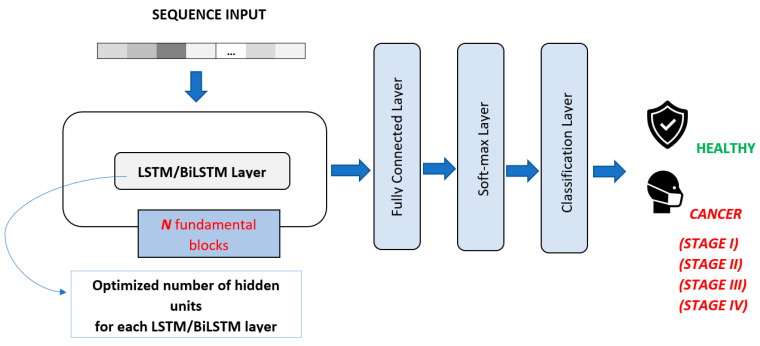
Proposed LSTM and BiLSTM architectures. (Illustrations by the authors).

In order to make a consistent comparison, the number of layers obtained for 1-D CNN was also used for LSTM and BiLSTM models. For LSTM and BiLSTM DL algorithms, the number of hidden units in each LSTM and BiLSTM layers was optimized by applying GA (Table 5 and Table 6). The general structure of the LSTM and BiLSTM architectures is shown in Figure 6.

For each of the DL algorithms, adaptive moment estimation (Adam) optimizer was utilized, and early stopping was applied to prevent overfitting. In addition, data shuffling was enabled before each training epoch.

### 3.4. Evaluation Criteria

In this study, different evaluation methods were used to test the performance of ML and DL models over the data. 

The first metric of this study was classification accuracy, which was calculated by the ratio of the number of correct predictions to the total number of samples/predictions (5). The accuracy will be high if most of the samples are correctly predicted.

(5) $$Accuracy=\frac{Number\;of\;Correct\;Predictions}{Total\;Number\;of\;Predictions}$$

The second metric that was used in this study is the Root Mean Square Error (*RMSE*), which is a widely used method to measure the gap between classification predictions and actual classes [73]. The equation to calculate *RMSE* can be seen in (6):

(6) $$RMSE=\sqrt{\frac{\sum_{n=1}^{N}{\left({\hat{r}}_{n}-r_{n}\right)}^{2}}{N}}$$

In Equation (6), ${\hat{r}}_{n}$ is predicted values, $r_{n}$ is observed values, and *N* is the number of observations. The results of RMSE are lower when the correct classification is employed. 

The Kappa statistic, which was presented by J. Cohen [74], is another metric that was used to evaluate the results of this study. Kappa statistic is a measure of the degree of agreement between two evaluations in a dataset [75]. Thus, it is expected that the classifiers with more overlapping prediction will generate higher Kappa values [76]. These values can be interpreted considering Table 7 according to Landis and Koch [77]. 

### 3.5. Statistical Tests

In this study, a statistical test—McNemar’s test—is employed to measure the statistical significance of the results. McNemar’s test [78] is a nominal variant of the Chi-square test which is utilized to analysis matched pairs of data. In this test, two different methods result in four possible outputs, which can be seen in Table 8. 

In Table 8, the number of times both algorithms failed or succeeded are represented by $N_{ff}$ and $N_{ss}$, respectively. These parameters are insignificant when comparing two algorithms performance in McNemar’s test. On the other hand, $N_{fs}$ and $N_{sf}$ indicate the number of times one algorithm succeeded and the other failed. These two parameters were used to calculate the *z* score (Equation (7)), which is the numerical representation of difference between performance of two algorithms.

(7) $$z=\frac{\left(\left|N_{sf}-N_{fs}\right|-1\right)}{\sqrt{N_{sf}+N_{fs}}}$$

If the *z* score is 0, then it can be interpreted as the two algorithms showing similar performance, which denotes insignificance. When the *z* score is a positive value, performances of algorithms differ from each other. In addition, it is important to note that z scores have corresponding confidence scores which can be seen in Table 9. 

## 4. Results and Discussion

### 4.1. Experimental Results

The synopsis of the proposed approach included the following steps:

(1) The obtained data that was composed of exRNA profiles/samples for healthy individuals and cancer patients was augmented by utilizing the SMOTE algorithm.
(2) Normalization was applied on the data to reduce the effect of outlier samples.
(3) A feature selection algorithm that calculates the information gain of each feature/attribute forming the data was applied. The algorithm ranked each attribute in descending order of value according to how informative it was.
(4) The samples with the different numbers of attributes according to their ranks were utilized as inputs to feed the canonical ML and DL algorithms to build models.
(5) The 10-fold cross-validation technique was utilized when building each model.
(6) To optimize the hyper-parameters of the DL architectures, the GA was utilized.
(7) The performance that each model achieved in terms of accuracy, RMSE, and Kappa statistic was determined.
(8) To reveal whether the performances of the models were statistically significant, McNemar’s test was applied.

**Figure 7:**
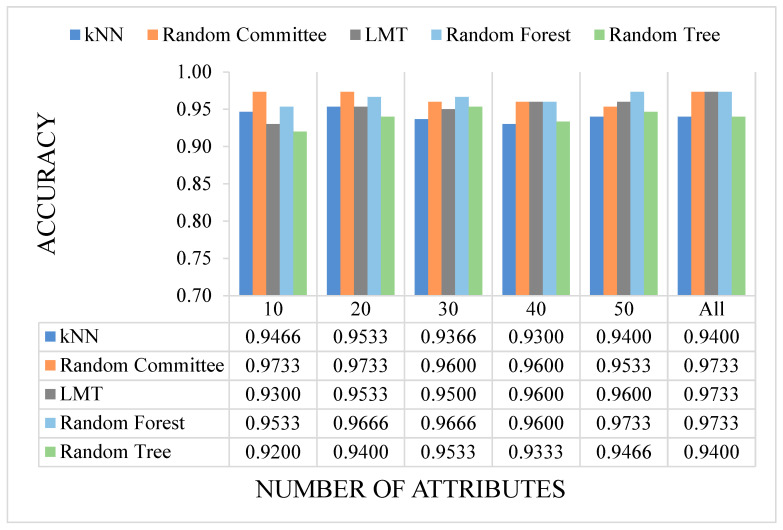
The accuracy results of the ML models on cancer prediction with different numbers of attributes. (Illustrations by the authors).

**Figure 8:**
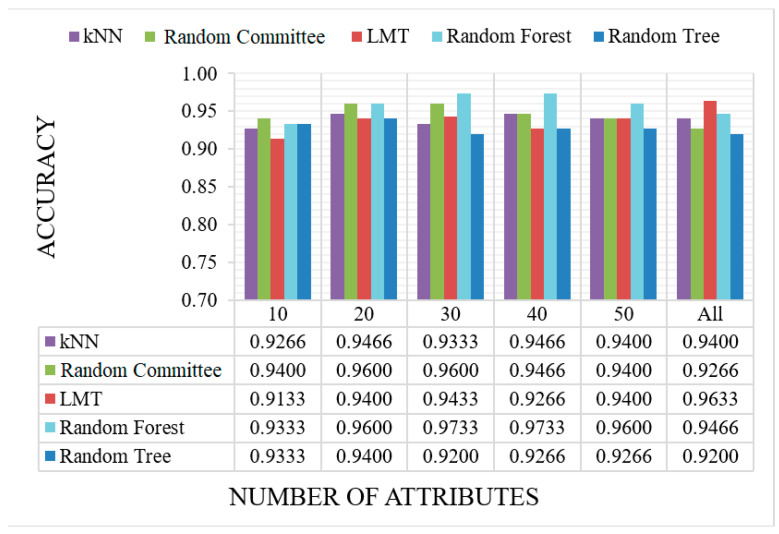
The accuracy results of the ML models on cancer stage classification with different numbers of attributes. (Illustrations by the authors).

**Figure 9:**
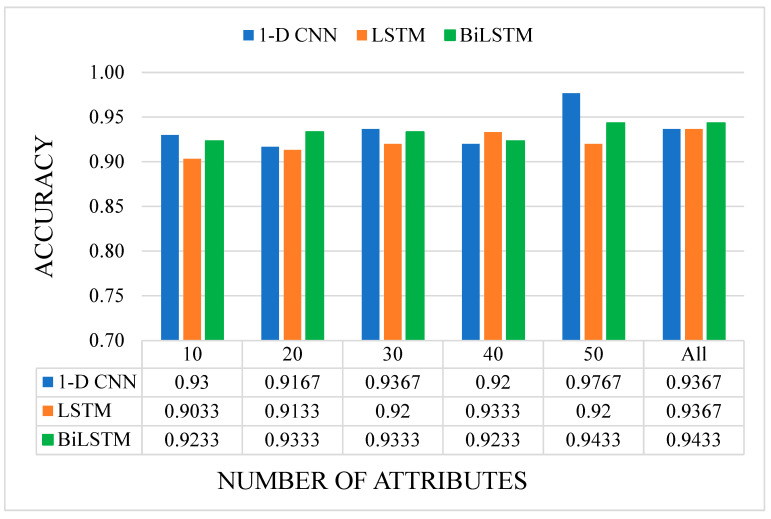
The accuracy results of the DL models on cancer prediction with different numbers of attributes. (Illustrations by the authors).

**Figure 10:**
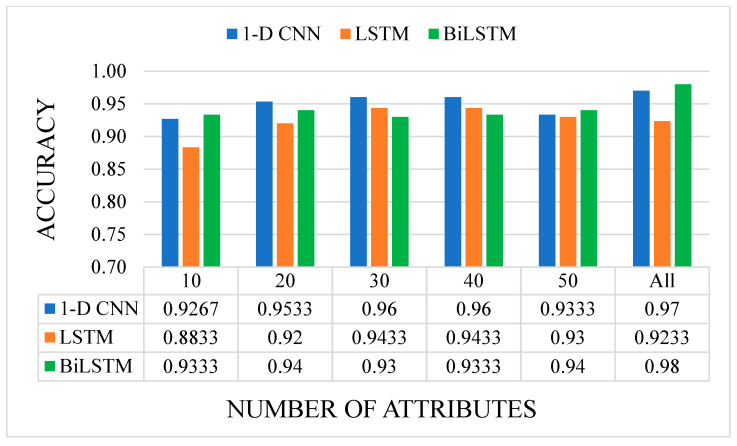
The accuracy results of the DL models on cancer stage classification with different numbers of attributes. (Illustrations by the authors).

In our present study, publicly available RNA-seq data of healthy individuals and colon cancer patients were downloaded and analyzed. We determined approximately 10 million raw sequence reads. Of these raw reads, approximately 40% were mapped into the reference RNA sequences. The data have been tested with five canonical ML and three DL algorithms mentioned before. All results are given as graphs in Figure 7, Figure 8, Figure 9 and Figure 10. In these figures, the *x*-axis corresponds to number of attributes, and the *y*-axis corresponds to achieved accuracy. The results of canonical ML algorithms can be seen in Figure 7 and Figure 8 for predicting cancerous samples and stage of the cancer, respectively. 

As seen in Figure 7, all five canonical ML methods yielded adequate results when predicting cancerous or healthy samples. All methods returned over 92% accuracy, which was the lowest result generated by RT method when 10 attributes were selected. Attribute selection was utilized to reduce the complexity of the models and shorten the training time. In general, selecting certain attributes did not improve the accuracy results. The RC, LMT, and RF methods provided the best results when all the attributes were used, while selecting attributes resulted in reduced accuracy of the LMT and RF methods. On average, RC and RF were the most successful methods when predicting the existence of cancer.

The results of the second test, which included predicting stages of cancer, can be seen in Figure 8. In this part of the study, the data included samples from healthy individuals and cancer patients at certain stages. The results wwere very promising, considering all five methods successfully classified at least 91% of the samples. Although there was no direct or inverse effect of attribute selection, the best result was achieved using RF when 30 and 40 attributes were selected out of 493 exRNA transcripts. Here, we can say that using the most informative 30 attributes was sufficient to classify the stage of the cancer. The most 50 informative attributes according to our feature selection method were: tRNA-Glu (also known as TRNAE3), hsa-miR-873-3p, hsa-miR-132-5p, hsa-miR-335-5p, hsa-miR-219a-5p, hsa-miR-139-3p, hsa-miR-22-5p, hsa-miR-409-3p, hsa-miR-152-3p, hsa-let-7e-5p, hsa-miR-425-5p, hsa-miR-543, hsa-miR-411-5p, hsa-miR-501-3p, hsa-miR-874-3p, hsa-miR-140-5p, hsa-miR-26a-1-3p, hsa-let-7i-3p, hsa-miR-660-5p, hsa-miR-378c, hsa-miR-19b-3p, hsa-miR-29c-3p, hsa-miR-370-3p, hsa-miR-130a-3p, hsa-miR-30c-5p, hsa-miR-363-3p, hsa-miR-30a-3p, hsa-miR-676-3p, hsa-miR-23b-3p, hsa-miR-767-5p, hsa-miR-145-3p, hsa-miR-1246, hsa-miR-885-5p, hsa-miR-125b-2-3p, hsa-miR-10b-5p, hsa-miR-1298-5p, hsa-miR-125a-3p, hsa-miR-339-3p, hsa-miR-23b-3p, hsa-miR-129-2-3p, hsa-miR-206, hsa-miR-34c-5p, hsa-miR-105-5p, hsa-miR-760, hsa-miR-330-5p, hsa-let-7d-5p, hsa-miR-10a-5p, hsa-miR-204-3p, hsa-miR-28-3p, and hsa-miR-99b-3p. As can be seen from the most informative 50 attributes, all attributes, except the first one, belong to the mature microRNA category. In general, the different methods had varying performances for changing numbers of attributes. RC achieved better performance if the number of attributes was relatively low, and as the selected number of attributes was increased, RF yielded the best results. Additionally, the LMT stood out as the best method when all attributes were used for evaluation. 

The results of DL algorithms can be seen in Figure 9 and Figure 10 for predicting cancer and stage of the cancer, respectively. According to the Figure 9, the highest accuracy was obtained by 1-D CNN model, while 50 attributes were utilized to train the convolutional neural network. In the LSTM model, the highest accuracy was obtained by including all attributes in the training, while in the BiLSTM model, the highest score was achieved by using both 50 and all attributes. It can be said that 50 attributes having the highest IG values were distinctive for binary classification with 1-D CNN model. 

According to Figure 10, for predicting the stage of the cancer, the highest accuracy was obtained by BiLSTM model with 98%, while all attributes were utilized to feed the classifier for predicting the stage of the cancer. The second highest accuracy rate of 97% was obtained with 1-D CNN model by enabling all attributes as the input of the classifier. However, the lowest accuracy rate of 88% was achieved with LSTM model by handling 10 attributes. Considering the utilization of all attributes, it was revealed that LSTM had the lowest accuracy rate once again. Nevertheless, LSTM model exceeded the 90% accuracy rate with all numbers of attributes, except when 10 attributes were selected as input.

The Kappa statistics and RMSE results of two experiments for canonical ML algorithms can be seen in Table 10 and Table 11. The results were obtained by using the dataset with all attributes. LMT algorithm showed the best performance regarding Kappa, but RC was the best algorithm considering RMSE on the state dataset. In Table 11, it can be clearly seen that LMT was superior on both the Kappa statistic and RMSE. These results support the accuracy graphs by showing the dominance of LMT on the dataset with all features. In addition, it is important to mention that all Kappa statistic values presented “Almost Perfect” agreement, considering Table 7.

The Kappa statistics and RMSE results of two experiments for DL algorithms in order to predict cancer and classify cancer stage can be seen in Table 12 and Table 13, respectively. The results were obtained by using the dataset with all attributes. According to Table 12, among the DL models, the best performance was achieved by BiLSTM. Compared with canonical ML models, DL models showed low performance in terms of Kappa value and RMSE. For both evaluation metrics, the best model among DL models showed 7% less performance than the best model among canonical ML models. However, the results were consistent with the accuracy performance and Kappa statistic values that indicated “Almost Perfect” agreement, considering Table 7. 

According to the Table 13, the highest values for both Kappa and RMSE were achieved by BiLSTM model. These achievements endorsed the accuracy performance by revealing the superiority of BiLSTM on classifying the stage of the cancer. In addition, it is important to mention that all Kappa statistic values indicated “Almost Perfect” agreement, considering Table 7. In addition, BiLSTM outperformed LMT in terms of Kappa but did not gain an advantage over RC and LMT in terms of RMSE. 

The results of McNemar’s test for canonical ML algorithms to predict cancer and classify cancer stage can be seen in Table A1 and Table A2, respectively. The results of McNemar’s test for DL algorithms to predict cancer and classify cancer stage can be seen in Table A3 and Table A4, respectively. In these tables the arrowheads show the superior classifier on the related dataset. The selected number of features are 10, 20, 30, 40, 50, and all respective attributes. Bold numbers (>1.96) indicate more than 95% confidence level for two-tailed predictions. The aforementioned tables show the statistical significance of the results by comparing two classifiers. In all tables, every sub-column represents the results for different numbers of attributes. In addition, the values over 1.96, which corresponds to the 95% confidence level for two-tailed predictions, are marked bold in the tables.

In Table A1, which shows the z scores of algorithms on the state dataset, it can be clearly seen that RF and RC outperformed other algorithms by having 18 and 17 arrowheads, respectively. In addition, there are three values exceeding 2.576, that represent a 99.5% confidence level. Two of these three values belong to RC, and the other one belongs to RF, which indicates the superiority of these classifiers. 

Z scores of algorithms on the stage dataset can be seen in Table A2. In this table, the RF classifier has 20 arrowheads, demonstrating more dominant performance than previous table. In addition, by having 14 arrowheads, the RC classifier performed second best algorithm on this dataset. In the table, seven values are marked in bold for RF classifier, which also indicates the superiority of this classifier. Another remarkable result is that RF classifier has four values representing 99.5% confidence level. It is also worth mentioning that LMT classifier has only seven arrowheads, and two of them have a confidence level of 99.5%. 

According to Table A3, it is revealed that 1-D CNN model outperformed other models by having nine arrowheads. Three of them are marked in bold representing 97.5% and 95% confidence levels for one-tailed and two-tailed predictions, respectively. In addition, one of them indicates 99.5% and 99% confidence levels for one-tailed and two-tailed predictions, respectively. Among these DL models, BiLSTM comes second with five arrowheads, whereas LSTM is the last with two arrowheads. It is worth mentioning that the 1-D CNN model showed a statistically significant difference versus the LSTM model for 10, 40, and all attributes to predict cancer.

In Table A4, the 1-D CNN model outperformed other DL models by having nine arrowheads to classify cancer stages. Four of them are marked in bold, representing 97.5% and 95% confidence levels for one-tailed and two-tailed predictions, respectively. In addition, one of them indicates 99.5% and 99% confidence levels for one-tailed and two-tailed predictions, respectively. Among these DL models, BiLSTM comes second with seven arrowheads, whereas LSTM is the last with one arrowhead. It is useful to emphasize that the 1-D CNN model showed a statistically significant difference with the highest confidence level versus the LSTM model while utilizing all attributes to classify cancer stage.

Our first hypothesis is validated according to the accuracy performance of canonical ML and DL models. For colon cancer prediction, the best accuracy was obtained by the 1-D CNN DL model with 97.67%, which outperformed other canonical ML models. Furthermore, for cancer stage classification, the best accuracy was obtained by the BiLSTM DL model with 98%, which outperformed other canonical ML models.

The second hypothesis is also validated by the feature selection algorithm that simply ranked the attributes according to the value of IG. It is revealed that 49 of the most informative 50 exRNA transcripts were miRNAs, and they belonged to the mature miRNA category.

The third hypothesis is validated for colon cancer prediction and cancer stage classification by canonical ML models. RC and RF models with 97.33% accuracy performance, fed by the most informative 10 and 50 exRNA transcripts, respectively, outperformed other ML models in cancer prediction. It is clearly seen that performance improvement could not be achieved when the number of exRNA transcripts used was increased. In cancer stage classification, RF model achieved the best accuracy performance with 97.33% by utilizing the most informative 30 and 40 exRNA transcripts. According to the accuracy performance of DL models for colon cancer prediction, the third hypothesis is also validated. The best accuracy performance was achieved with 97.67% by the 1-D CNN model utilizing only the most informative 50 exRNA transcripts. However, in cancer stage classification, the third hypothesis is invalidated by the accuracy performance of DL models. The best accuracy performance was achieved with 98% by the BiLSTM model utilizing all exRNA transcripts. On the other hand, this result is consistent with the findings of Yuan et al.’s study [18]. According to that study, as the stage of the disease progresses, the number of small non-coding RNAs (including miRNA, piwiRNA, and siRNA) increases. Therefore, the best accuracy performance can be expected by utilizing all exRNA transcripts that include other miRNAs, piwiRNAs, and siRNAs to classify cancer stage.

### 4.2. Practical Applicability

**Figure 11:**
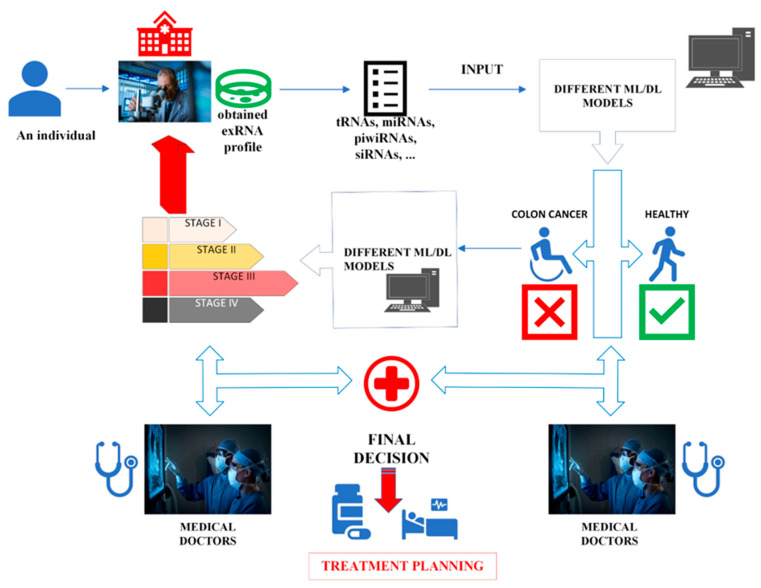
Practical applicability of the proposed approach. (Illustrations by the authors).

Our proposed approach can have an applicability in practice. It can be utilized for both diagnosis and prognosis. Our approach alone should not be considered to diagnose colon cancer. The main goal is to assist medical doctors as a second opinion during diagnosis/prognosis and to speed up the process of treatment planning (Figure 11). 

A scenario for the practical applicability of the approach can be as follows:

- An individual with health complaints applies to a health institution.
- A diagnosis is made after a medical doctor’s examination and modern medical tests (healthy or colon cancer).
- The medical doctor may misdiagnose or seek a second opinion, as the symptoms will not be the same in every individual.
- The medical doctors may disagree on a diagnosis, as they may also come from different medical traditions.
- At this stage, the approach we propose can become a part of the medical process.
- After the exRNA profile of the individual is obtained, it is given to the canonical ML and DL models as input.
- According to the results of the different models, the medical doctors can agree on a diagnosis or confirm their diagnosis.
- It becomes important to determine the stage of the disease after the diagnosis.
- Our approach can be utilized not only for diagnosing colon cancer but also for determining the stage of the cancer.
- If the disease has not progressed to the final stages, early detection of the cancer accelerates treatment planning and improves the patient’s likelihood of recovery and quality of life.

The advantage of our proposed approach is that re-obtaining a patient’s exRNA transcript values—without requiring procedures that affect the patient physically and psychologically—is sufficient to determine whether the disease is progressing.

Our approach can be applied to other types of cancer as well. All that is required is to obtain the exRNA profiles from healthy individuals and patients with a specific cancer. Later, canonical ML and DL models can be obtained and optimized from the data. Additionally, the models can be retrained with new inputs and become more robust and less error-prone. 

Considering the workload on medical doctors in the COVID-19 pandemic, the efficacy of our approach can be better understood. If our approach is utilized, it can be provided that doctors make consistent decisions supported by artificial intelligence and shorten the time they spend per patient. Therefore, medical doctors can have time to spare for resting and preparing for other patient appointments.

## 5. Conclusions

In this study, five canonical ML and three DL models were utilized to predict whether an individual has colon cancer and to classify the stage of the cancer. We used RNA-seq data of EVs, which was deposited at NCBI. EVs have drawn attention for early diagnosis of cancer. They carry DNA, RNA, protein, and metabolites between cancer cells for cellular communication. Therefore, evaluation of molecular components in vesicles provides detailed information about cancer progression. In recent years, transcriptome structure of vesicles has been analyzed frequently to find biomarkers. We focused on total transcriptome structure with ML and DL models to find new perspectives which could be used in clinical practice.

One of the remarkable results of the study is that although hyper-parameters of canonical ML models were not optimized, they showed as high accuracy performance as DL models did for predicting cancer and classifying cancer stage. However, DL models achieved the best accuracy results by applying a meta-heuristic search algorithm, namely GA, resulting in a longer model training duration.

Input data were normalized between 0 and 255 to create the 1-D CNN model in cancer prediction. The highest accuracy rate was obtained with this method. From this point of view, we consider that this method can also be used in the prediction of other cancer types. 

Another important aspect of the study is that BiLSTM model outperformed both canonical ML models and other DL models in terms of accuracy of classifying cancer stages. This can be explained by the learning ability of bidirectional long-term dependencies in sequence data through the layers in the BiLSTM architecture. Therefore, we determined that BiLSTM can reveal the relationships among various types of RNA within samples.

Despite the limited amount of data available, DL and ML architectures achieved promising results. This situation proves that the proposed approach has potential for building an efficient prediction framework for colon cancer studies. Several shortcomings exist in the study. Only GA is considered for hyper-parameter estimation of DL models. In future studies, other meta-heuristic optimization algorithms, such as particle swarm optimization, ant colony, and gray wolf optimization, could be employed to compare the performances. This will increase validity and impact of the proposed approach. Since DL architectures have a high computational need to train data, this need can hinder the implementation performance of the proposed approach. In future studies, this issue can be investigated by using parallel computing tools and advanced Graphics Processor units. Finally, the results can be improved furthermore with a larger volume of data and by integrating canonical ML models with DL models to obtain ensemble classifiers. 

## Figures and Tables

**Table 1:** Default parameters for ML algorithms. (Illustrations by the authors).

Canonical ML Algorithm	Default Parameters
kNN	Euclidean distance is used, and k is determined as 7 by grid search.
LMT	Minimum number of instances at which a node can be split is 15.
RT	No limit is determined for maximum depth of the tree.
RC	Number of iterations is 10.
RF	Number of trees is 100.

**Table 2:** 1-D CNN architecture according to the size of the input. (Illustrations by the authors).

Number of Attributes	Number of Convolutional and Max Pooling Layers
10	4, 4
20	5, 5
30	5, 5
40	6, 6
50	6, 6
493 (All)	9, 9

**Table 3:** Filter parameters of each convolutional layer for cancer prediction. (Illustrations by the authors).

Number of Attributes	Filter size in Convolutional Layers											
	Number of Filters in Convolutional Layers											
10	3		5				3				2	
	17		28				32				96	
20	11		7		3				3		2	
	94		56		124				46		88	
30	21		5		3				2		3	
	46		84		93				104		76	
40	13		12	9			3			2	4	
	96		123	111			74			86	52	
50	3		7	8			1			3	1	
	38		65	24			19			76	112	
All	15	21	18	9		11		5		4	7	3
	124	111	76	68		45		102		49	88	96

**Table 4:** Filter parameters of each convolutional layer for cancer stage classification. (Illustrations by the authors).

Number of Attributes	Filter Size in Convolutional Layers											
	Number of Filters in Convolutional Layers											
10	7		5				3				3	
	22		34				31				77	
20	7		5		5				2		2	
	41		52		64				103		90	
30	13		5		2				3		1	
	66		45		78				82		126	
40	11		7	3			5			1	2	
	71		99	102			111			87	103	
50	22		8	5			4			3	3	
	102		59	76			84			105	125	
All	19	23	11	7		5		4		4	6	2
	93	84	114	122		105		77		70	95	106

**Table 5:** Optimized number of hidden neurons for cancer prediction. (Illustrations by the authors).

Number of Attributes	Number of Hidden Neurons (LSTM)											
	Number of Hidden Neurons (BiLSTM)											
10	102		125				147				122	
	151		107				134				128	
20	125		106		140				137		105	
	100		134		106				102		117	
30	139		104		104				182		177	
	106		145		121				110		129	
40	129		124	118			103			109	141	
	144		107	112			135			144	126	
50	174		153	101			129			182	168	
	150		109	112			165			178	190	
All	172	195	190	108		134		137		195	183	177
	188	192	146	200		200		125		147	154	169

**Table 6:** Optimized number of hidden neurons for cancer stage classification. (Illustrations by the authors).

Number of Attributes	Number of Hidden Neurons (LSTM)											
	Number of Hidden Neurons (BiLSTM)											
10	107		130				132				125	
	101		142				134				131	
20	152		161		139				128		111	
	122		150		145				142		126	
30	157		144		135				164		182	
	118		127		139				144		162	
40	133		121	127			200			181	196	
	177		172	148			162			154	160	
50	199		190	174			155			163	171	
	182		176	170			143			181	193	
All	194	155	167	153		144		162		170	168	188
	175	187	200	149		141		190		166	155	178

**Table 7:** The interpretation of Kappa values [77].

Kappa Statistic	Strength of Agreement
<0.00	Poor
0.00–0.20	Slight
0.21–0.40	Fair
0.41–0.60	Moderate
0.61–0.80	Substantial
0.81–1.00	Almost Perfect

**Table 8:** Possible results of algorithms. (Illustrations by the authors).

	Algorithm A Failed	Algorithm A Succeeded
Algorithm B failed	$N_{ff}$	$N_{sf}$
Algorithm B succeeded	$N_{fs}$	$N_{ss}$

**Table 9:** Confidence levels corresponding to z scores for one- and two-tailed predictions [62].

*z* Score	One-Tailed Prediction	Two-Tailed Prediction
1.645	95%	90%
1.960	97.5%	95%
2.326	99%	98%
2.576	99.5%	99%

**Table 10:** Kappa statistics and RMSE values of algorithms on cancer prediction with all attributes. (Illustrations by the authors).

Method	Kappa Statistic	RMSE
Random Forest	0.9388	0.2185
Random Committee	0.9388	**0.1683**
Random Tree	0.867	0.2449
k-Nearest Neighbor	0.8788	0.2304
Logistic Model Tree	**0.9397**	0.1821

**Table 11:** Kappa statistics and RMSE values of algorithms on cancer stage classification with all attributes. (Illustrations by the authors).

Method	Kappa Statistic	RMSE
Random Forest	0.9311	0.1747
Random Committee	0.9057	0.1258
Random Tree	0.8976	0.1789
k-Nearest Neighbor	0.9230	0.1542
Logistic Model Tree	**0.9528**	**0.1247**

**Table 12:** Kappa statistics and RMSE values of deep learning algorithms on cancer prediction with all attributes. (Illustrations by the authors).

Method	Kappa Statistic	RMSE
CNN	0.8564	0.2517
LSTM	0.8579	0.2517
BiLSTM	**0.8709**	**0.2380**

**Table 13:** Kappa statistics and RMSE values of deep learning algorithms on cancer stage classification with all attributes. (Illustrations by the authors).

Method	Kappa Statistic	RMSE
CNN	0.9320	0.1732
LSTM	0.8313	0.2769
BiLSTM	**0.9548**	**0.1414**

## Data Availability

Data are contained within the article. The data presented in this study are available in [18].

## Abbreviations

ML	Machine Learning
RNA-seq	RNA sequence
CNN	Convolutional Neural Network
LSTM	Long Short-Term Memory
BiLSTM	Bidirectional Long Short-Term Memory
DL	Deep Learning
EV	Extracellular vesicle
GEO	Gene Expression Omnibus
RPM	Reads per million
SMOTE	Synthetic Minority Over-Sampling Technique
Min–max normalization formula	$x^{\prime }=\frac{x-\min\left(X\right)}{\max\left(X\right)-min\left(X\right)}\left(new\_max\left(X\right)-new\_min\left(X\right)\right)+new\_min\left(X\right)$
min(X)	The current minimum value
max(x)	The current maximum value
new_min(x)	New minimum value
new_max(y)	New maximum value
IG	Information gain
Information gain formula	$IG\left(C,X\right)=Entropy\left(C\right)-\sum_{x\in X}\frac{X_{x}}{X}*Entropy\left(X_{x}\right)$
Entropy formula	$Entropy=-\sum_{i=1}^{c}P\left(x_{i}\right)log_{2}P\left(x_{i}\right)$
C	Target or class
X	Attribute vector
x	Each value of the attribute vector X
c	The number of the cases of the target or briefly the number of classes
$P\left(x_{i}\right)$	The probability of a value occurring in the target data.
kNN	k-Nearest Neighbors
LMT	Logistic Model Tree
RT	Random Tree
RC	Random Committee
RF	Random Forest
RNN	Recurrent Neural Networks
SVM	Support Vector Machine
AUC	Area Under Curve
DT	Decision Tree
PD	Parkinson’s disease
PLSDA	Partial Least Squares Discriminant Analysis
XGBoost	eXtreme Gradient Boosting
MLP	Multilayer Perceptron
1-D CNN	One-dimensional Convolutional Neural Network
GA	Genetic algorithm
PCA	Principal Component Analysis
Crossover formula	$g_{i}\left(C_{c}\right)=\{\begin{array}{c} g_{i}\left(C_{p2}\right),\;b_{i}=0\\ g_{i}\left(C_{p1}\right),\;b_{i}=1 \end{array}$
$g_{i}$	The *i*th gene
$C_{c}$	Child chromosome
$C_{p1}$ and $C_{p2}$	Parent chromosomes
$b_{i}$	The *i*th value in the random binary vector.
Adam	Adaptive moment estimation
Accuracy formula	$Accuracy=\frac{Number\;of\;Correct\;Predictions}{Total\;Number\;of\;Predictions}$
RMSE	Root Mean Square Error
RMSE formula	$RMSE=\sqrt{\frac{\sum_{n=1}^{N}{\left({\hat{r}}_{n}-r_{n}\right)}^{2}}{N}}$
${\hat{r}}_{n}$	Predicted values
$r_{n}$	Observed values
N	Number of observations
z score formula	$z=\frac{\left(\left|N_{sf}-N_{fs}\right|-1\right)}{\sqrt{N_{sf}+N_{fs}}}$
$N_{fs}$, $N_{sf}$	Number of times one algorithm succeed and other failed

## Appendix A

**Table A1:** Z scores of ML algorithms on cancer prediction. (Illustrations by the authors).

	RC						RT						kNN						LMT					
**RF**	**↑ 1.58**	**↑ 0.7**	**← 0.31**	**0**	**← 1.58**	**0**	**← 2.4**	**← 2.02**	**← 0.86**	**← 1.75**	**← 2.02**	**← 2.12**	**← 0.31**	**← 1.5**	**← 2.66**	**← 1.94**	**← 2.4**	**← 2.02**	**← 1.37**	← 1.06	← 1.1	0	← 0.75	0
RC							**← 3.75**	**← 2.4**	← 0.31	← 1.56	← 0.23	**← 2.4**	**← 2.02**	**← 2.04**	← 1.54	← 1.94	← 0.86	**← 2.47**	**← 2.91**	**← 2.04**	← 0.45	0	↑ 0.23	0
RT													↑ 1.75	↑ 0.75	← 1.03	0	← 0.23	↑ 0.23	↑ 0.48	↑ 0.75	0	↑ 1.56	↑ 0.75	↑ 1.91
kNN																			← 0.97	0	↑ 0.7071	↑ 1.74	↑ 1.06	↑ 2.02

**Table A2:** Z scores of ML algorithms on cancer stage classification. (Illustrations by the authors).

	RC						RT						kNN						LMT					
**RF**	**↑ 0.31**	**0**	**← 1.06**	**← 2.47**	**← 1.33**	**← 1.58**	**0**	**← 1.58**	**← 3.75**	**← 3.47**	**← 2.4**	**← 1.75**	**← 0.40**	**← 0.86**	**← 2.75**	**← 1.75**	**← 1.58**	**← 0.31**	**← 1.33**	← 1.58	**← 2.21**	**← 3.06**	← 1.33	↑ 1.03
RC							← 0.31	← 1.58	**← 2.75**	← 1.58	← 1.5	← 0.31	← 1.06	← 0.75	← 1.75	0	0	↑ 0.86	← 1.75	**← 2.04**	← 1.10	← 1.33	0	**↑ 2.77**
RT													← 0.40	← 0.23	↑ 1.06	**↑ 2.04**	↑ 0.86	↑ 1.33	← 1.58	0	↑ 1.66	0	↑ 0.75	**↑ 2.75**
kNN																			← 0.86	← 0.26	↑ 0.48	← 1.17	0	↑ 1.3

**Table A3:** Z scores of DL algorithms on cancer prediction. (Illustrations by the authors).

	1-D CNN						LSTM						BiLSTM					
**1-D CNN**							**← 2.75**	**← 0.92**	**← 0.46**	**← 2.41**	**← 1.81**	**← 2.00**	**← 0.35**	**↑ 0.92**	0	0.60	**←** 1.77	**←** 1.79
LSTM													**↑ 2.43**	↑ 1.03	← 0.29	← 0.24	↑ 0.92	↑ 1.66

**Table A4:** Z scores of DL algorithms on cancer stage classification. (Illustrations by the authors).

	1-D CNN						LSTM						BiLSTM					
**1-D CNN**							**← 1.67**	**← 2.01**	**← 1.66**	**← 2.02**	**← 1.55**	**← 3.47**	**↑ 1.03**	**← 0.29**	**← 2.12**	← 1.81	↑ 0.35	↑ 1.16
LSTM													**↑ 2.31**	**↑ 2.09**	0	← 0.29	↑ 1.65	**↑ 3.18**
